# The Effect of Process Parameters on the Performance of the RFSSW of ZK61M-T5 Magnesium Alloy

**DOI:** 10.3390/ma18204743

**Published:** 2025-10-16

**Authors:** Di Jiang, Ling Ji, Hongfeng Wang, Xiaole Ge

**Affiliations:** 1 College of Mechanical and Electrical Engineering, Huangshan University, Huangshan 245041, China; 2 School of Logistics, Linyi University, Linyi 276000, China

**Keywords:** magnesium alloy, refill friction stir spot welding, ultimate lap shear force, microstructure

## Abstract

This study investigates refill friction stir spot welding (RFSSW) of 2 mm thick ZK61M-T5 magnesium alloy. Sound joints were obtained at rotational speeds of 1000 rpm and 1500 rpm with welding speeds of 30–50 mm/min. At 1000 rpm, micro-pores appeared at the sleeve-affected zone bottom, exhibiting a full-fillet fracture and a maximum ultimate lap shear force (ULSF) of 8.3 kN. Increasing the speed to 1500 rpm eliminated the pores and changed the fracture mode to partial fillet, but reduced the ULSF to 7.7 kN. Higher welding speeds caused the Hook feature to shift from upward to downward. Compared with the base material, grains in the thermomechanically and sleeve-affected zones were refined, while those in the pin-affected zone coarsened with increasing rotational speed. Overall, higher rotational speed increased heat input and mitigated internal defects, but enhanced annealing and Zr segregation, leading to reduced mechanical performance.

## 1. Introduction

Magnesium alloys, as one of the lightest structural metal materials, have garnered extensive attention and found broad application in aerospace, automotive, electronics, and medical industries due to their outstanding properties. With a density only two-thirds that of aluminum, they contribute significantly to weight reduction in transportation equipment, thereby improving fuel efficiency and extending operational range [1,2]. Additionally, they have good vibration damping and anti-vibration properties, making them suitable for automotive components such as seat frames, steering wheels, and door frames, thereby enhancing safety and comfort [3,4].

Despite their excellent specific strength, magnesium alloy structural components present considerable challenges in joining different parts. Conventional mechanical fastening techniques such as bolting and riveting are relatively inferior to welding in terms of joint efficiency. Therefore, commonly used welding methods include tungsten inert gas welding (GTAW), metal inert gas welding (MIGW), laser welding, and electron beam welding. However, these fusion welding methods introduce high thermal input, which poses the risks of oxidation and combustion of magnesium alloys. Additionally, the resulting welds are prone to porosity and other defects. In this context, friction stir spot welding (FSSW), as a solid-state welding technology, shows great potential for magnesium alloy applications [5,6,7].

FSSW leaves a keyhole in the weld zone, which limits significant improvement in joint strength. This issue is overcome by refill friction stir spot welding (RFSSW), an advanced derivative technology. RFSSW employs axially movable tools comprising a stir pin and a stir sleeve that can perform axial movement to complete the welding process, producing a weld zone that is smooth and flush with the surface, resulting in higher joint strength. As such, it has become a current mainstream research direction. Since this technology is a derivative of friction stir welding, a process predominantly applied for aluminum alloy welding, RFSSW has primarily been used in aluminum alloys. Jiang D et al. conducted extensive research on 6061-T6 aluminum alloy, revealing the influence of stir tool diameter, pin-to-sleeve ratio, and process parameters on the performance of the spot-welding zone [8,9,10].

Due to the lower plasticity and faster heat conduction of magnesium alloys compared to aluminum alloys, their spot-welding formability is generally poorer. This makes the application of RFSSW to magnesium alloys more challenging and underscores the importance of researching RFSSW for magnesium alloys. Zhang H et al. [11] used RFSSW to join 2 mm AZ91D-H24 magnesium alloy plates, studying the effect of penetration depths of 2.0 mm and 2.5 mm on the tensile shear performance of the joints. They found that the penetration depth significantly affected the height of the ‘Hook’, which in turn reduced the tensile shear performance of the joints. The optimal tensile shear strength achieved was 6.4 kN under a rotational speed of 1200 rpm, a penetration depth of 2.5 mm, and a penetration rate of 0.9 mm/s. Liu Z et al. [12] applied RFSSW to join 1 mm thick AZ31 magnesium alloy sheets, investigating the metallurgical characteristics, microstructure, texture, and mechanical response of the spot weld joints. The highest lap shear strength (2.6 kN) was achieved at a rotation speed of 1500 rpm, an insertion depth of 1.4 mm, and a welding speed of 0.2 mm/s. The grain size in the stir zone decreased with reduced rotation speed and insertion depth. Additionally, significant strain-induced deformation was observed in the spot weld during tensile deformation. L.C. Campanelli et al. [13] conducted RFSSW on 2 mm thick AZ31 magnesium alloy, with a detailed analysis of crack propagation. Their findings indicated that the height of the ‘Hook’ reduces the ULSF (ultimate lap shear force) of the joint in the spot-welding zone. Under a rotational speed of 1500 rpm and a penetration depth of 2.75 mm, an optimal ULSF of 4.87 kN was obtained. Fu B et al. [14] explored RFSSW of cast magnesium alloys and reported relatively low shear strength of the spot weld joints. To improve performance, they applied differential rotational RFSSW (DR-RFSSW) for spot welding, which promoted discontinuous dynamic recrystallization and mitigated deformation incompatibility between the weld nugget zone and the thermomechanically affected zone. As a result, the shear strength increased by 50%, reaching 6.8 kN.

Research on RFSSW of magnesium alloys mainly focuses on the AZXX series, which are commonly used in the manufacturing of structural components in low-temperature environments. These alloys exhibit a significant decrease in strength when the service temperatures exceed 120 °C. In contrast, ZK61M magnesium alloy is a typical high-strength, heat-treatable material that retains favorable mechanical properties below 200 °C, and is often used in aerospace, defense, and transportation sectors. However, it is prone to issues such as strength reduction and significant softening at the weld joints after welding [5,12,15,16,17,18,19,20]. To address these challenges, this study investigates RFSSW of 2 mm thick ZK61M magnesium alloy in the T5 condition. The key research points include the locations where internal defects occur in the spot-welding zone, as well as the ‘Hook’ morphology that affects ULSF. Ultimately, the ULSF of the spot-welding zone and the annealing softening phenomenon are obtained, with the aim of expanding the application scenarios of this series of magnesium alloys and improving their welding quality.

## 2. Test Methods

**Figure 1:**
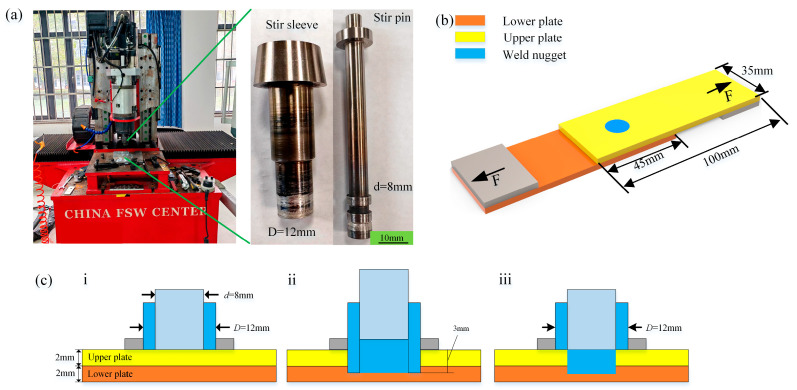
Diagrams of RFSSW and the tensile shear test. (**a**) RFSSW equipment and its stirring tool; (**b**) diagram of tensile shear test of the RFSSW sample; (**c**) diagram of the process flow for RFSSW; i: Welding Preparation Phase, ii: Welding penetration stage, iii: Welding completion stage.

The base material used in this study was ZK61M-T5 magnesium alloy (Jinseng Metal Co., Ltd. in Guangzhou City, China), whose fundamental properties are listed in Table 1 and Table 2. These data were provided by Kunyu Metal Products Co., Ltd. in Dongguan, China (a metal materials manufacturer) and verified by the authors through experimental measurements to ensure consistency with the performance testing methods applied to the subsequent spot-welding zone. A schematic of the RFSSW equipment (Saifus Technology Co., Ltd. in Beijing, China) and the dimensions of the stirring tool is presented in Figure 1a. As shown, the tool consists of a stirring sleeve with a diameter of 12 mm and a stirring pin with a diameter of 8 mm. To better understand the effect of process parameters on the spot-welding zone, rotational speeds of 1000 and 1500 rpm were selected, which were controlled by a servo motor control and exhibited fluctuations within 0.5%. Moreover, the radial oscillation of the stirring tool was limited to 0.03 mm. The welding speed, limited by equipment performance, was set to 30, 40, and 50 mm/min. The welding speed refers to the axial movement speed of the stirring sleeve, which remained constant during both the penetration stage and the refill stage. Additional parameters included a dwell time of 2 s, a welding pressure of 7 kN, a maximum spindle torque of 25 N·m, a clamping ring diameter of 25 mm, and a maximum insertion depth of the stirring sleeve of 3 mm, as illustrated in Figure 1c. ULSF of the welded samples was measured on a tensile shear testing machine, with ASTM E8 Standard Test Methods for Tension Testing of Metallic Materials adopted as its test standard [21]. To ensure more accurate measurement and aligned loading, a spacer was placed on each side of the sample to ensure that the load was as perpendicular as possible to the spot-welding zone, as shown in Figure 1b. For each parameter combination, five tests were performed, and the average ULSF was taken, with the tensile shear speed kept constant at 1.5 mm/min.

## 3. Results and Discussion

**Figure 2:**
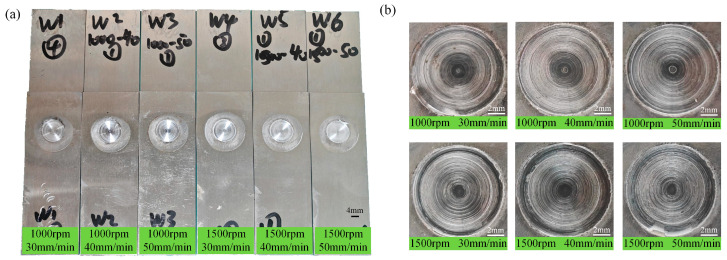
Macroscopic morphology of spot-welding zone. (**a**) Image of spot-welding samples under different processes; (**b**) Spot welding zone local image of the spot-welding zone under different processes.

**Figure 3:**
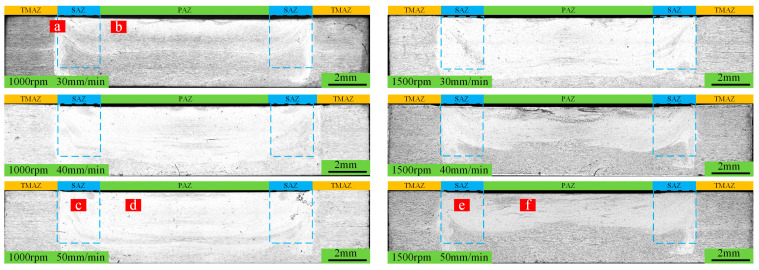
Macroscopic cross-sectional morphology of spot welding under different process parameters. PAZ (pin-affected zone), TMAZ (temperature- and mechanical-affected zone), SAZ (sleeve-affected zone). The blue line represents the boundary of SAZ.

Figure 2 and Figure 3, respectively, show the macroscopic morphology and cross-sectional morphology of the spot-welding zone under different process parameters.

**Figure 4:**
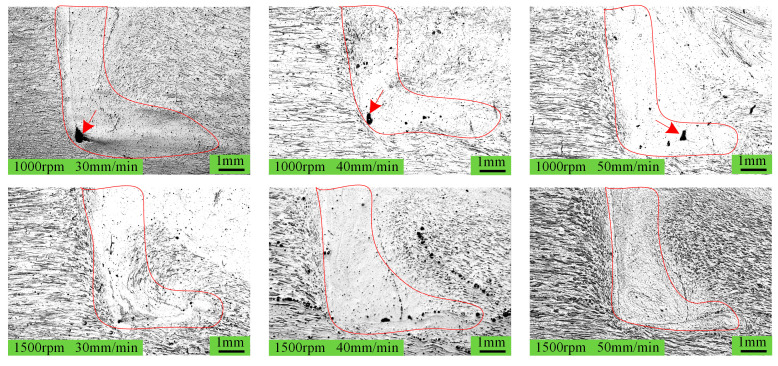
Morphology of the toe region under different welding parameters.

Figure 2a shows an overall image of the spot-welding zone, and it is evident that the sample has not undergone significant bending deformation. Figure 2b clearly displays the surface morphology of the weld nugget, and overall, no surface defects are observed. The results reveal that these process parameters can achieve RFSSW. This absence of major defects is also evident in the cross-section of the spot-welding zone, as shown in Figure 3. However, under higher magnification, micro-pores are observed at a rotation speed of 1000 rpm, as indicated by the arrows in Figure 4.

These defects occur at the bottom of the sleeve-affected zone and appear unaffected by variations in welding speed. Analysis suggests that the bottom of the sleeve-affected zone is the region farthest from the spot weld center, subject to complex metal flow in both horizontal and vertical directions. Regardless of the direction, the plastically flowing material encounters significant resistance at this location and stops moving, thus leaving a flow trace very similar to a ‘boot’ shape, as outlined by the red lines in Figure 4. This flow pattern also makes the root of the ‘boot’ the least accessible region for refill metal. At a rotation speed of 1000 rpm, the generated heat is significantly less than at 1500 rpm, which results in poorer plastic material flowability within the spot-welding zone, ultimately leading to the formation of micro-pores at the ‘boot’ root.

The morphology of the ‘Hook’ affects the overall mechanical performance of the spot-welding zone, especially as it often serves as the initiation site for crack formation under tensile shear load and then propagates along the ‘Hook’ [22,23,24].

**Figure 5:**
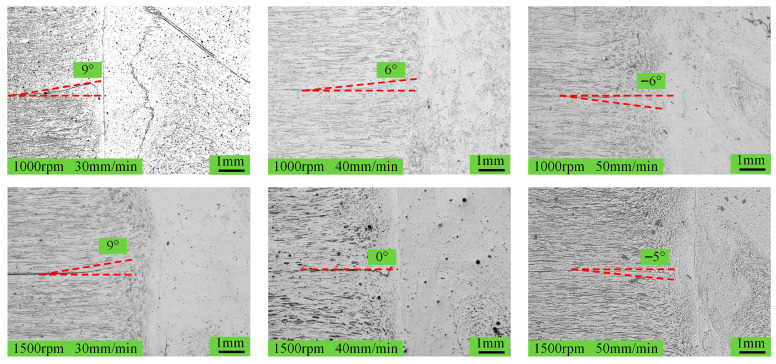
Morphology of “Hook” under different process parameters.

**Figure 6:**
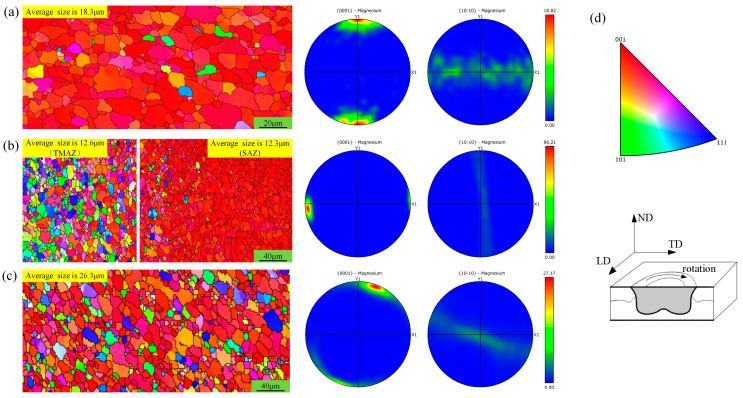
Grain distribution in the spot-welding zone and base metal. (**a**) Grain distribution of ZK61M base material; (**b**) sampling location as shown in area a of Figure 3; (**c**) sampling location as shown in area b of Figure 3; (**d**) test direction as indicated.

Regardless of the rotation speed, the ‘Hook’ transitions from an upward to a downward orientation as the welding speed increases. However, it is noticeable that when the rotation speed is 1500 rpm, the degree of curvature of the ‘Hook’ is less pronounced. This behavior is attributed to the fact that the ‘Hook’ is in the thermomechanically affected zone, and its morphology is mainly influenced by the metal flow form in this zone. At lower welding speeds, the heat input at the spot-welding zone is greater, which effectively enhances the metal softening, allowing the stirrer pin to penetrate easily without causing significant deformation in the thermomechanically affected zone. During the refill stage, as the stirrer pin moves upward, the longer thermal exposure further softens the thermomechanically affected zone, resulting in upward movement of the stirrer pin and an upward curvature of the ‘Hook’. In contrast, when the welding speed increases, the heat input in the thermomechanically affected zone decreases, increasing the resistance to the penetration of the stirrer pin. At this point, the penetration of the stirrer pin inevitably causes the metal in the thermomechanically affected zone to deform plastically downward together. During the refill stage, the thermal effect becomes greater but still relatively weak, which restricts the ability of the stirrer pin to cause deformation in the thermomechanically affected zone, resulting in a downward curvature of the ‘Hook’. This phenomenon becomes pronounced as the welding speed increases. When the rotation speed is 1500 rpm, although the heat input is considerably higher, it fails to fully reverse the bending tendency of the “Hook,” leading to a geometry with diminished curvature, as shown in Figure 5. To further analyze the grain distribution within the spot-welding zone, Figure 6 shows the grain images of the base metal and the spot-welding zone (1000 rpm, 30 mm/min).

The ZK61M magnesium alloy used in this study was cold-rolled, and the initial distribution direction and orientation intensity are shown in Figure 6a. Numerous studies have reported that the grains in the spot-welding zone are significantly refined and those in the TMAZ remain unchanged or become larger [8,9,10,11]. However, this study reveals a contrasting behavior: compared to the grains in the base material, they are smaller in both the TMAZ and SAZ but larger in the PAZ, as shown in Figure 6b and Figure 6c, respectively. Additionally, the grain orientation in the sleeve-affected zone rotates 90 degrees compared to that in the base material, accompanied by a stronger orientation intensity. The grain orientation in the pin-affected zone rotates by approximately 15 degrees compared to that in the base material, with an enhanced orientation intensity. Analysis suggests that the sleeve-affected zone is subjected to shearing and compressive forces from the stirring sleeve, which aligns with the rolling thickness direction of the base material but is perpendicular to the rolling direction. The effect of the stirring sleeve is similar to the thermal rolling effect in the thickness direction and the horizontal direction, thus resulting in a 90-degree deviation in the orientation direction and a stronger orientation intensity. The stirring pin produces a similar effect to the stirring sleeve, and the difference lies in its smaller diameter, which results in less severe horizontal shearing force and a moderate orientation direction of about 15 degrees.

**Figure 7:**
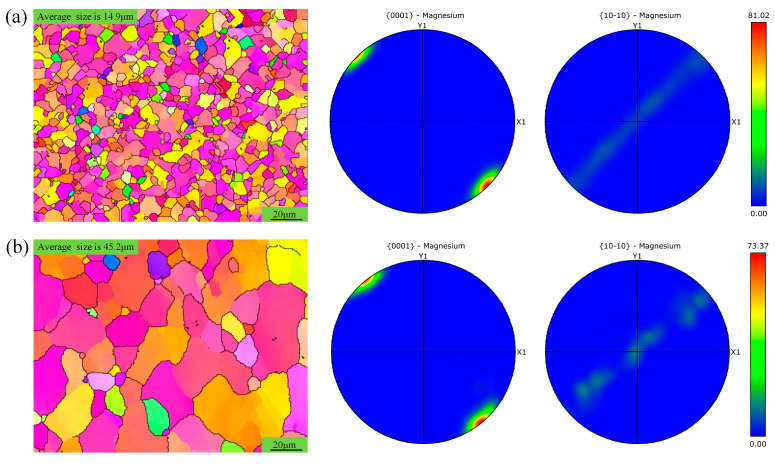
Grain distribution of sleeve-affected zone under different rotational speeds. (**a**) Grain distribution at 1000 rpm and 50 mm/min for sampling location as shown in area c of Figure 3; (**b**) grain distribution at 1500 rpm and 50 mm/min for sampling location as shown in area e of Figure 3.

To further understand the effect of rotational speed on the grain structure within the spot-welding zone, the grain distribution in the SAZ was analyzed at different rotational speeds, as shown in Figure 7.

The grain orientation in the SAZ remains highly consistent across different rotational speeds, generally exhibiting a 45-degree deviation from that of the base material, with a significantly higher orientation intensity. The findings are similar to the observations in the aforementioned Figure 6. However, when the rotational speed is 1500 rpm, the grains in the SAZ are noticeably larger than those of the base material, indicating that increased rotational speed enhances the heat input and promotes significant grain growth.

**Figure 8:**
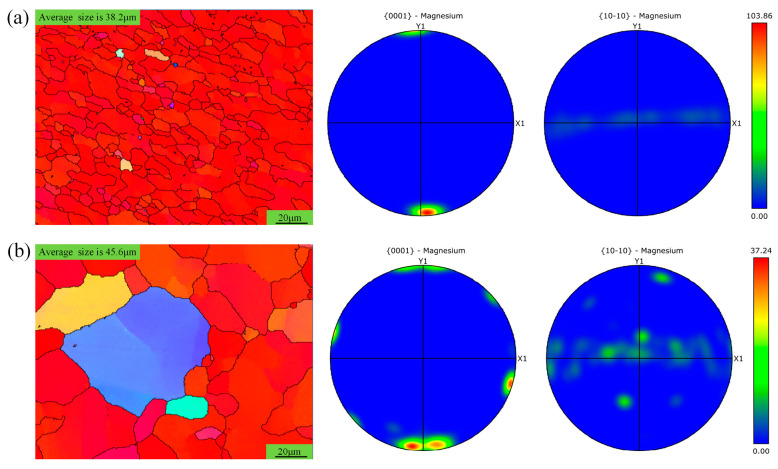
Grain distribution of pin-affected zone under different rotational speeds. (**a**) Grain distribution at 1000 rpm and 50 mm/min for sampling location as shown in area d of Figure 3; (**b**) grain distribution at 1500 rpm and 50 mm/min for sampling location as shown in area f of Figure 3.

For the pin-affected zone under different rotational speeds, the grain orientation only deviates slightly from that of the base material, as shown in Figure 8. At a rotational speed of 1000 rpm, the orientation strength is about 5.5 times stronger than that of the base material, while at 1500 rpm, it is approximately 2 times stronger. In comparison, lower rotational speeds result in relatively finer grain sizes, but they still exceed those of the base material. This can stem from the smaller diameter of the stirring pin, which limits its ability to refine grains in the stirring sleeve. Additionally, higher heat input at elevated rotational speeds promotes grain growth. In terms of grain orientation, the deviation from the base material is less than 15 degrees, as observed in Figure 6. This is because the higher welding speed at this time results in a shorter shear action duration, which is insufficient to significantly shear and compress the metal in the pin-affected zone. However, the compression displacement in the thickness direction is consistent, indicating a higher speed, thus resulting in a stronger grain orientation strength.

**Figure 9:**
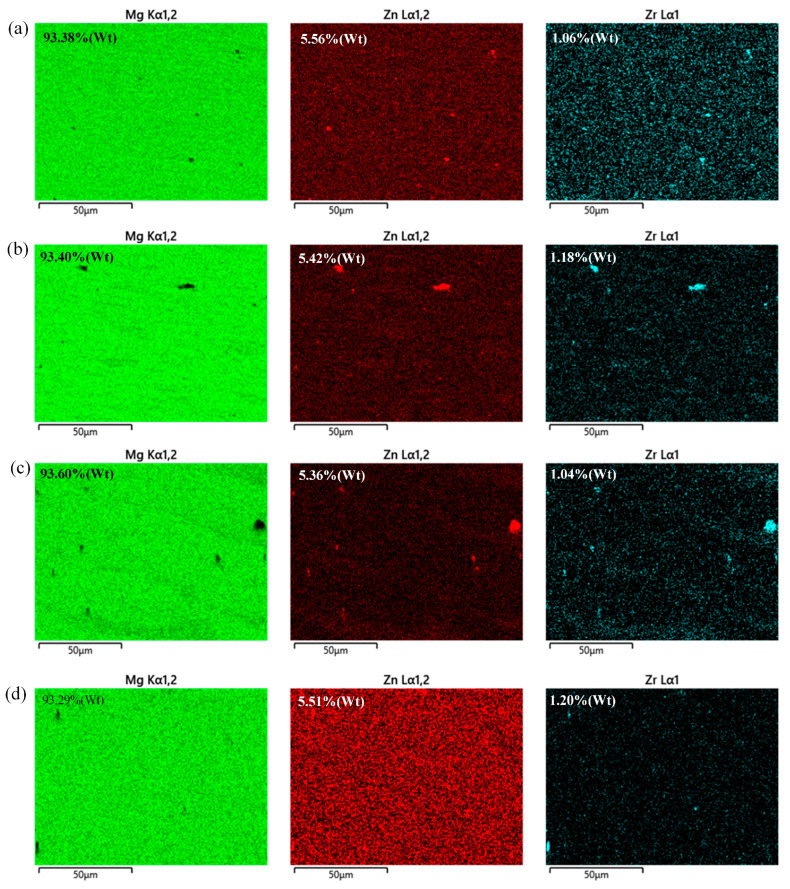
EDS images of the spot-welding zone at different rotational speeds. (**a**) EDS images at 1000 rpm and 50 mm/min for sampling location as shown in area c of Figure 3; (**b**) EDS images at 1000 rpm and 50 mm/min for sampling location as shown in area d of Figure 3; (**c**) EDS images at 1500 rpm and 50 mm/min for sampling location as shown in area e of Figure 3; (**d**) EDS images at 1500 rpm and 50 mm/min for sampling location as shown in area f of Figure 3.

As presented in Figure 9, a lower content of Zr but a higher content of base material are observed in the SAZ compared to that in the PAZ. This fully demonstrates that higher temperature input enhances the solubility of Zr but slightly affects the content of Zn. Under different rotational speeds, these trends are consistent, which also indicates the effective effect of generated temperatures on the solubility of Zr at 1000 rpm or 1500 rpm.

**Figure 10:**
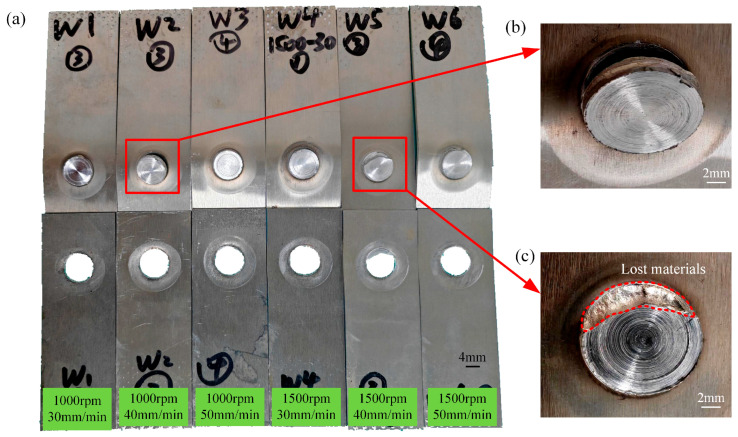
Morphology of tensile shear fracture under different process parameters for spot welding. (**a**) Fracture images of spot welding zones under different parameters; (**b**) Protrusion fracture; (**c**) Partial protrusion fracture.

**Figure 11:**
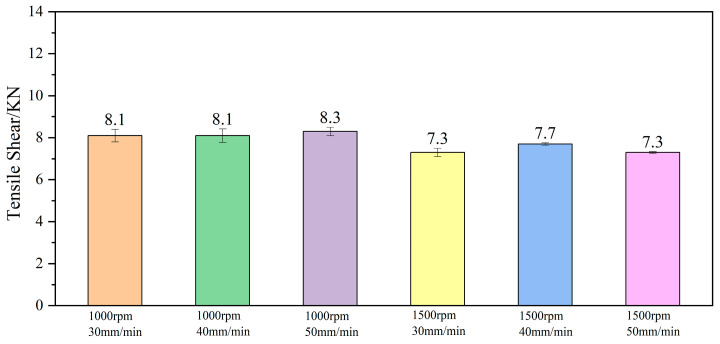
ULSF of spot-welding zone under different process parameters.

At a rotation speed of 1000 rpm, the tensile shear fracture in the spot-welding zone is basically complete protrusion fracture, as shown in Figure 10b. However, at a rotation speed of 1500 rpm, it is mostly partial protrusion fracture, especially at a welding speed of 40 mm/min, as shown in Figure 10c. The welding speed has little effect on the tensile shear level of the spot-welding zone, while the rotation speed exerts a significant impact. At a rotation speed of 1000 rpm, the average ULSF is 1.1 times higher than that at 1500 rpm, as illustrated in Figure 11.

Analysis suggests that neither the ‘Hook’ morphology nor the defect at the root of the ‘boot’ mentioned above appear to significantly affect the ULSF of the spot-welding zone. In fact, joints produced under conditions where these defects were present demonstrated comparatively higher strength. Additionally, the degree of ‘Hook’ curvature did not change the complete fracture mode. The rotational speed was identified as the decisive factor affecting ULSF, mainly due to the associated higher heat input leading to grain growth, which consequently reduced the mechanical performance.

**Figure 12:**
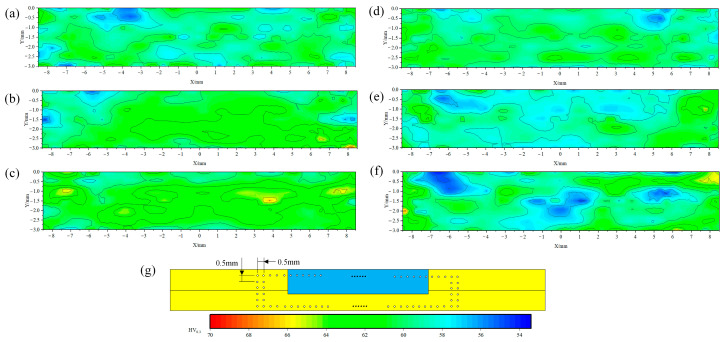
Microhardness distribution in the spot-welding zone under different process parameters: (**a**) 1000 rpm, 30 mm/min; (**b**) 1000 rpm, 40 mm/min; (**c**) 1000 rpm, 50 mm/min; (**d**) 1500 rpm, 30 mm/min; (**e**) 1500 rpm, 40 mm/min; (**f**) 1500 rpm, 50 mm/min. (**g**) Hardness test results.

The spot-welding zones under different process parameters were polished to a mirror finish. Hardness testing was conducted in accordance with the provisions specified in ASTM E384 standard using a Vickers hardness tester equipped with a square-based pyramid indenter [25]. A load of 0.3 kg was applied and held for 10 s. The diagonal lengths of the resulting indentations were measured optically to determine the hardness values. Each sample was measured three times, and the average value was calculated and adopted as the final result. The hardness distribution is shown in Figure 12, with the measurement positions and intervals detailed in Figure 12g. The test results indicate that the sleeve-affected zones are relatively obvious low-hardness zones, which are pronounced at higher rotational speeds. At the same rotational speed, increasing the welding speed also yields better microhardness distribution, which is consistent with the aforementioned lower Zr content in the sleeve-affected zone. Overall, the average hardness in the spot-welding zone seems lower than that of the base material, with only small areas achieving better hardness values. Analysis suggests that this observation can be attributed to the intense squeezing and fracturing by the stirring tool, which introduces significant thermal input and plastic deformation, leading to annealing and uneven Zr distribution. Although these factors generally reduce hardness, localized grain refinement in certain areas increases Zr concentration, resulting in isolated regions of elevated hardness.

## 4. Conclusions

This study completed RFSSW on 2 mm ZK61M magnesium alloy and investigated the ULSF, grain distribution status, and hardness distribution under rotational speeds of 1000 rpm and 1500 rpm and welding speeds of 30 mm/min, 40 mm/min, and 50 mm/min. The following conclusions were drawn:

(1) At a rotational speed of 1000 rpm, micro-pores were observed at the bottom of the sleeve-affected zone regardless of welding speed. In contrast, no such defects occurred at 1500 rpm. This indicates that higher rotational speeds can provide better plastic flowability for the magnesium alloy, resulting in a defect-free spot-welding zone.
(2) Regardless of the rotational speed, increasing the welding speed caused the ‘Hook’ to change from an upward to a downward orientation, with reduced curvature observed at 1500 rpm. The grain size in the thermomechanically affected zone and sleeve-affected zone was finer than those of the base material, but the grains in the pin-affected zone were coarser and increased in size with the increase in rotational speed. Additionally, the grain orientation in the sleeve-affected zone was rotated by 90 degrees compared to the base material, with stronger orientation, whereas the grain orientation in the pin-affected zone was rotated by about 15 degrees. These results confirm that higher rotational speeds introduce greater heat input, causing the grains in the pin-affected zone to become larger.
(3) At a rotational speed of 1000 rpm, the tensile shear fracture was basically complete protrusion fracture, while it was mostly partial protrusion fracture at 1500 rpm in the pin-affected zone. Such a difference was due to the varying bending morphologies of the ‘Hook’. At a rotational speed of 1000 rpm, the average ULSF L was 1.1 times higher than that at 1500 rpm. The spot-welding speed and internal defects had little effect on this finding because higher rotational speeds result in greater heat input, causing grain growth and reducing its performance. This was also evident in the microhardness distribution in the spot welding zone, where greater heat input led to pronounced annealing and uneven distribution of Zr, thus reducing the overall mechanical performance.

## Figures and Tables

**Table 1:** Chemical composition of ZK61M magnesium alloy.

Each Element’s Composition (wt) %							
Zn	Zr	Al	Si	Fe	Cu	Ni	Mg
5.45	0.64	0.01	0.01	0.008	0.007	0.004	Others

**Table 2:** Mechanical and physical properties of ZK61M magnesium alloy.

Tensile Strength/MPa	Elongation/%	Hardness/HV_0.3_	Average Grains/μm
320	9	75	18.3

## Data Availability

The original contributions presented in this study are included in the article. Further inquiries can be directed to the corresponding author.
